# The investigation of *WNT6* and *WNT10A* single nucleotide polymorphisms as potential biomarkers for dental pulp calcification in orthodontic patients

**DOI:** 10.1371/journal.pone.0288782

**Published:** 2023-08-11

**Authors:** Iago Ramirez, Christian Kirschneck, Alice Corrêa Silva-Sousa, Peter Proff, Leonardo S. Antunes, Marilisa C. L. Gabbardo, Daniela Silva Barroso de Oliveira, Manoel D. Sousa-Neto, Flares Baratto-Filho, Erika C. Küchler

**Affiliations:** 1 School of Dentistry, University of São Paulo (FORP-USP), Ribeirão Preto, Brazil; 2 Department of Orthodontics, University of Regensburg, Regensburg, Germany; 3 School of Dentistry, Tuiuti University from Paraná, Curitiba, Paraná, Brazil; 4 Department of Specific Formation, Fluminense Federal University, Nova Friburgo, Brazil; 5 School of Health Sciences, University Positivo (UP), Curitiba, Brazil; 6 School of Dentistry, Federal University of Alfenas (UNIFAL-MG), Alfenas, Brazil; 7 Department of Dentistry, University of Joinville Region (Univille), Joinville, SC, Brazil; Medical University of South Carolina, UNITED STATES

## Abstract

The aim of this study is to evaluate if single nucleotide polymorphisms (SNPs) in *WNT6* and *WNT10A* are associated with the risk of dental pulp calcification in orthodontic patients. This cross-sectional study followed the “Strengthening the Reporting of Genetic Association Studies” (STREGA) guidelines. Panoramic radiographs (pre- and post-orthodontic treatment) and genomic DNA from 132 orthodontic patients were studied. Dental pulp calcification (pulp stones and/or pulp space narrowing) was recorded in upper and lower first molars. The SNPs in *WNT6* and *WNT10A* (*rs7349332*, *rs3806557*, *rs10177996*, and *rs6754599*) were assessed through genotyping analysis using DNA extracted from buccal epithelial cells. The association between pulp calcification and SNPs were analyzed using allelic and genotypic distributions and haplotype frequencies (p<0.05). Prevalence of dental pulp calcification was 42.4% in the 490 studied molars. In the genotypic analysis, the SNPs in *WNT10A* showed a statistically significant value for molar calcification (p = 0.027 for *rs1017799*), upper molar calcification (p = 0.040 for *rs1017799*) (recessive model), and molar calcification (p = 0.046 for *rs3806557*) (recessive model). In the allelic distribution, the allele C of the SNP *rs10177996* in *WNT10A* was associated with molar calcifications (p = 0.042) and with upper first molar calcification (p = 0.035). Nine combinations of haplotypes showed statistically significant value (p<0.05). The findings of this study indicates that SNPs in *WNT10A* and *WNT6* are associated with dental pulp calcification in molars after orthodontic treatment and may be considered as biomarkers for dental pulp calcification.

## Introduction

Teeth are composed of three different types of calcified tissues: enamel, dentin, and cementum. Dentin is the most abundant and the first developed hard dental tissue, which is responsible to determine the general shape, mechanical properties, and functions of teeth [1]. Dentin is similar to bone in its matrix protein composition, but unable to respond such as the bone in case of damage and does not go under remodeling [2]. The dentin combined with the dental pulp tissue, generally called the dentin-pulp complex, is capable to induce repair instead of remodeling under trauma [3]. The calcification of the dental pulp, partially or completely, is one of the ways that the dentin-pulp complex reacts by repairing under damage. This mechanism directly affects clinical approaches, such as endodontic treatment [4].

Dental pulp calcifications are discrete or diffuse calcified structures present in any portion of the pulp, either in the chamber or canals [5]. They are detected usually in radiographic examination and can be observed as radiopaque areas of variable sizes and shapes [6]. Dental pulp calcification may be categorized into 2 types: pulp stones and narrowing of the radicular pulp space. The key differences between them are the clinical presentation, in which the pulp stone is presented as well-defined radiopaque masses and the narrowing as a diffuse calcification along the dentin walls, with or without projections [5].

The exact etiology of the dental pulp calcification is unknown. Some factors have been involved in the risk of the occurrence of dental pulp calcifications, such as pulp degeneration processes, idiopathic factors, age, poor circulatory supply, inductive interaction between epithelium and pulp tissue, and also orthodontic tooth movements [7]. A recent systematic review concluded that orthodontic force triggers a sequence of biological responses that can affect the dental pulp, leading to an increase in pulp calcification [8].

Individual genetic predisposition might also play a role in the individual susceptibility to dental pulp calcification formation [9]. It is possible that genetic variations could be involved in pulp calcification risk [10]. According to a recent review, at least 300 genes are involved in the secretion, maturation, and regulation of dentin [11]. Different pathways, related to proteins and extracellular organic matrix, mineralization, and growth factors are involved in dentinogenesis, but the exact role of each pathway is not been fully elucidated so far. Not restricted to dentin secretion and mineralization, the mechanism behind the stress response and dentin bridge formation under damage, to date, remains to be fully comprehended. The wingless-type MMTV integration site family (WNT) is a commonly studied pathway, participating in both ways of dentinogenesis and dentin stress response. The WNT pathway is known to regulate multiple stages of tooth development and post-eruption maintenance [12]. WNT pathway is known as crucial in the physiological and pathological processes of dental pulp tissues, modulating many cellular events in dental pulp cells via the canonical and non-canonical pathways [13]. There are evidences of WNT signaling the survival of odontoblasts and the continued production of dentin [3]. Also, there are strong suggestions that it is related to the dentin matrix formation and mineralization [13]. An *in vitro* study using dental pulp cells showed the positive effects of activation of WNT/β-catenin signaling on odontoblast differentiation, suggesting the potential role of the WNT in pulp reaction to injuries [14]. A study with mice for dentin injury model observed that WNT-responsive odontoblasts secrete new dentin after superficial dental injury [15]. Also, a recent systematic review suggested that orthodontic tooth movement changes the expression of genes in the dental pulp [16]. The activation of WNT signaling pathway during orthodontic tooth movement was also previously observed [17].

From the members of the WNT family, *WNT10A* is a gene involved in the development of ectodermal derivates, including teeth [18]. It was previously associated with dental-related syndromic and non-syndromic conditions [19], and some of its single nucleotide polymorphisms (SNPs) were already associated with dentin matrix alterations and dental anomalies [20, 21].The SNP rs3806557 is located in a conserved promoter flanking region in *WNT10A*, which are frequently associated with regulatory effects on gene expression and was associated with dental phenotypes [22]. Two intronic SNPs rs7349332 and rs10177996 in *WNT10A* were also strongly associated with dental phenotype in a previous study [21]. *WNT6* is another important gene involved in tooth development, associated with dental papilla cell migration and differentiation and dentin bridge formation [20]. An intronic SNP rs6754599 in *WNT6* was associated with a dental-related phenotype [21]. These studies [21, 22] suggest the important role of rs6754599, rs3806557, rs7349332 and rs10177996 in dental-related tissues and cells. So, the aim of this study is to evaluate if SNPs in *WNT6* and *WNT10A* are associated with the risk of dental pulp calcification in orthodontic patients. The main hypothesis of this study is that the SNPs rs6754599, rs3806557, rs7349332 and rs10177996 are involved in the risk of dental pulp calcification on orthodontic patients, and that those SNPs are potential biomarkers for this phenotype.

## Methods

### Sample description and dental pulp calcification assessment

The study was previously approved by the Human Ethics Committee of the University of Regensburg (approval number: 19-1549-101). The project was performed according to the Helsinki Declaration, and informed consent was obtained from the legal guardians and/or the participants. The study followed the Strengthening the Reporting of Genetic Association study (STREGA) statement checklist [23], which is an Extension of the STROBE Statement for reporting genetic association studies.

Panoramic radiographs (pre- and post-orthodontic treatment) and genomic DNA of orthodontic patients were studied. The sample was composed only by patients that underwent orthodontic treatment with fixed appliances, from a previous study [24]. The inclusion criteria were: patients who underwent orthodontic treatment without any extraction of the maxillary and mandibular first and second premolar and molar treatments, without any loss of teeth due to caries lesions or trauma, without dental agenesis of any permanent teeth (excluding third molars) and without severe malocclusion that require further orthognathic surgery. Cases with low quality radiograph (poor angulations, improper exposures, or faulty processing) were excluded.

Any patients with a positive history of medical conditions in the anamnesis, syndromes, and oral cleft were excluded from the study to prevent any bias due to systemic disorders.

For the purpose of this study, all first upper and lower permanent molars were evaluated for the presence of dental pulp calcification, partially or completely. Firs molars (upper and lower molars) were assessed due their role as anchorage during orthodontic treatment. Molars presenting deep-extensive carious lesions or restorations; the presence of crown, bridges, or endodontic treatment; dental anomalies that compromise the radiographic evaluation, open apex, and confirmed story of dental trauma [25] were not included in the analysis. Molars with dental pulp calcification identified previously to orthodontic treatment were also excluded from the analysis.

The analysis was performed by one endodontic examiner, after training and calibration with a senior endodontic expert with 25 years of clinical experience. Inter-observer (0.81) and intra-observer (0.94) concordance were assessed by Cohen’s Kappa, with a very good agreement. All of the radiographs that satisfied the study requirements were examined digitally and in a dark room [25]. Each radiograph was analyzed using Windows Photo Viewer software, for Windows 10 (Microsoft Corporation, Redmont, WA, USA), on a 14-in Lenovo 81V7S00100 monitor (Lenovo PC International, Beijing, China) with 1,360×768-pixel resolution. The examiner manipulated the images to improve their contrast and brightness to access the clearest images in the analyzed areas [26].

A molar was recorded as having a dental pulp calcification when masses of calcified tissue present on the level of the pulp chamber and roots were observed radiographically. Therefore, pulp calcification was determined when the first molar presented pulp stones in pulp chambers and/or canals, and/or narrowing of the radicular pulp space also in the chambers and/or canals. Pulp stones were defined as definite radiopaque masses inside pulp chambers or canals, and the narrowing was defined as a severe reduction in the volume/size of the pulp chamber and/or root canals as reported in Kaplan et al. [26]. A decision was made to consider whether the molar has developed or not dental pulp calcification after orthodontic treatment based on these radiographic parameters comparing panoramic radiographs before and after orthodontic treatment.

The influence of orthodontic variables was also taken into consideration. The orthodontic data regarding age of treatment (beginning and end of treatment), treatment duration (in years) and type of skeletal malocclusion were collected. The skeletal malocclusion was defined as Class I (0 <ANB < 4), Class II (ANB ≥ 4), and Class III (ANB ≤ 0) according to ANB Steiner angle.

### Genotyping of the selected SNPs

The selection of the genes was initially based on the screening of previously published studies suggesting that *WNT10A* and *WNT6* are involved in tooth development, dentin formation, and odontoblast activation [20–22, 27], suggesting their role in dental-related tissues and cells. Subsequently, SNPs in these genes were screened from the dbSNP database (http://www.ncbi.nlm.nih.gov/snp/) and SNPinfo (http://snpinfo.niehs.nih.gov/), based on the following criteria: previous association with dental phenotypes, MAF (minor allele frequency) ≥ 10% in the global population and previously associated with calcification conditions. The characteristics of the selected SNPs are presented in Table 1.

**Table 1:** Characteristics of the selected SNPs.

Gene	Chromosome	SNP	Base Change	Global MAF*	Description
*WNT10A*	2q35	**rs7349332**	T/C	0.127	Associated with tooth morphology [21] and dental anomalies [27].
		**rs3806557**	A/G	0.322	Associated with tooth development and agenesis [22].
		**rs10177996**	C/T	0.210	Associated with tooth formation [21].
*WNT6*	2q35	**rs6754599**	G/C	0.376	Associated with crown size [21].

* MAF means Minor Allele Frequency

The genotyping analysis was performed with genomic DNA extracted from buccal epithelial cells using two cytobrushes placed in extraction solution (Tris-HCl 10 mmol/L, pH 7.8; EDTA 5 mmol/L; SDS 0.5%, 1 mL), as previously described [28]. The DNA was extracted using an established protocol [29]. Spectrophotometry (Nanodrop 1000, Thermo Scientific, Wilmington, DE, USA) was used to determine the concentration and purity of the DNA.

Four SNPs in *WNT10A* and *WNT6* were evaluated in this study and are described in Table 1. The genotyping was blindly performed using the Taqman™ method for real-time PCR in the Mastercycler®ep realplex-S thermocycler, Eppendorf AG (Hamburg, Germany). Additionally, 10% of the sample was genotyped twice and an agreement of 100% was observed. Patients with not enough DNA, or DNA samples that failed to be genotyped were excluded from the further analyses.

### Statistical analysis

A sample-size calculation was performed assuming a prevalence of dental pulp calcification in the first molars similar to 50% [30]. The alpha = 5% and power = 80% were used as parameters. Based on the average minor allele frequency of the selected SNPs, and assuming a 25% difference, a minimum sample of 120 was required.

For statistical analysis, narrowing of the radicular pulp space and pulp stones were grouped as one main group: ‘pulp calcification’ and was compared with ‘control’ (no calcification). Chi-square and t-test were used to compare orthodontic variables according to groups. PLINK was used to analyze the allelic and genotypic distributions, Hardy-Weinberg Equilibrium (HWE) and haplotype frequencies. Chi-square was used to compare allele and genotype distribution according to the groups. Odds ratio (OR) and confidence intervals of 95% were also obtained. The haplotype frequencies were compared using chi-square or Fisher’s Exact test. The significance level was determined as *p*<0.05.

## Results

**Fig 1:**
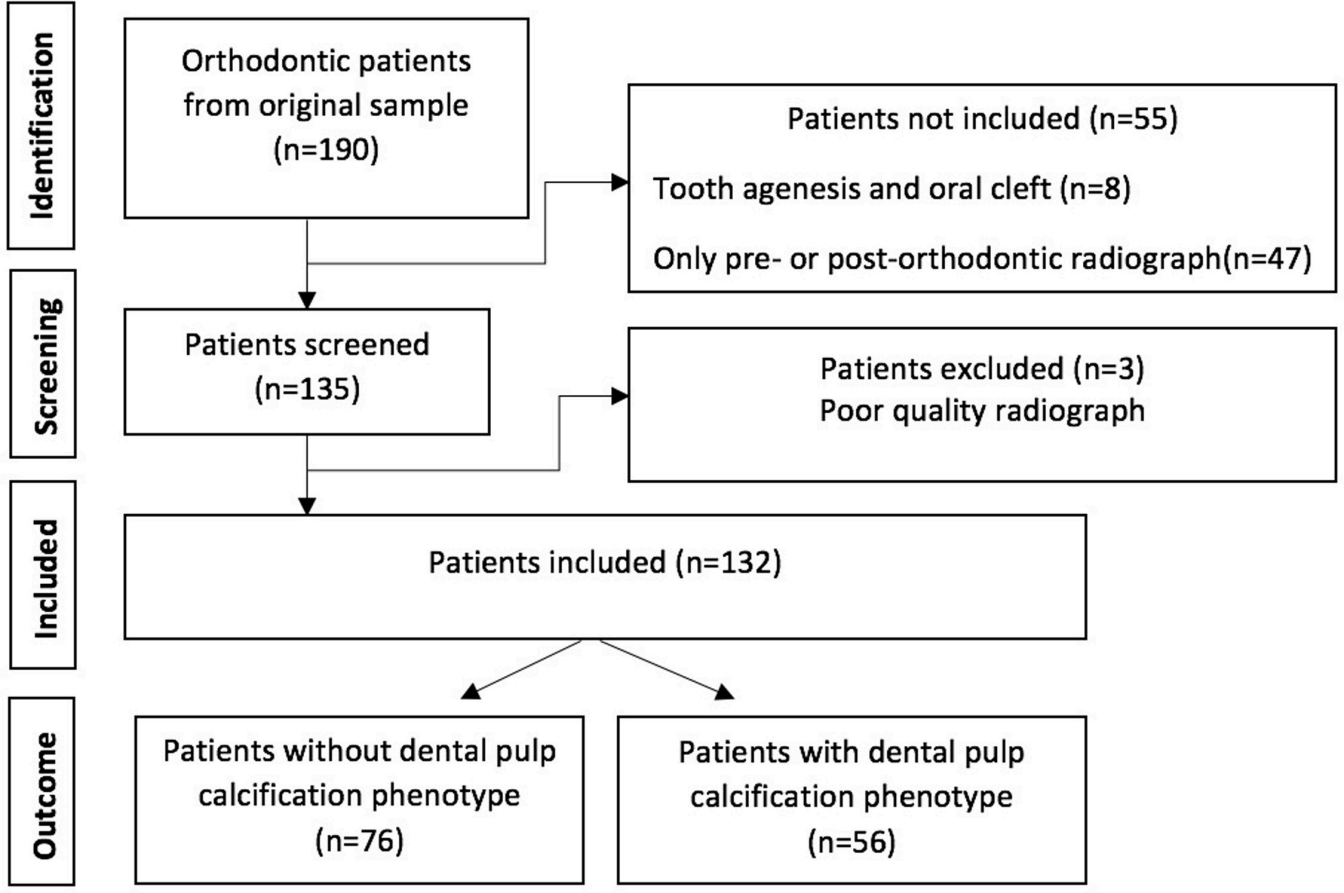
Flowchart according to STREGA statement checklist to the sample selection.

The flowchart of this study is presented in Fig 1, initially with 190 patients screened. Finally, a total of 490 molars from 132 patients (71 males, and 61 females) were studied. The prevalence of dental pulp calcification after orthodontic dental movement in at least one first molar (upper and/or lower molar) was 42.4% (n = 56), in at least one upper molar was 37.9% (n = 50), and in at least one lower molar was 20.5%, (n = 27). Orthodontic fixed treatment duration, as well as age at the beginning and end of the orthodontic treatment were compared among pulp calcification and control (no calcification) groups. Age and duration were not associated with pulp calcification (p>0.05). These results are shown in Table 2.

**Table 2:** Description of the sample.

Characteristic of the orthodontic fixed treatment	Mean (SD) in years				
	Control (no calcification)	Calcification groups			p-values
		All molars (upper + lower)	Upper molars	Lower molars	
Treatment duration	1.84 (0.61)	1.71 (1.31)	1.74 (1.36)	1.81 (0.68)	>0.05
Age at the beginning of treatment	14.34 (1.83)	14.91 (2.60)	15.00 (2.70)	14.85 (2.41)	>0.05
Age at the end of treatment	16.09 (1.94)	16.71 (2.66)	16.82 (2.76)	16.78 (2.22)	>0.05

Note: SD means standard deviation. T test was used. All comparisons were performed using no calcification as control.

The type of skeletal malocclusion according to the groups (pulp calcification and control) is presented in the Table 3. Skeletal malocclusion was not associated with dental pulp calcification.

**Table 3:** Skeletal malocclusion according to pulp calcification status.

Skeletal malocclusion	N (%)			
	Control (no calcification)	Calcification groups		
		All molars (upper + lower)	Upper molars	Lower molars
Class I	34 (44.7)	26 (46.4)	11 (44.7)	24 (48.0)
Class II	35 (46.1)	29 (51.8)	15 (55.6)	26 (52.0)
Class III	7 (9.2)	1 (1.8)	1(3.7)	0 (0.0)
p-value	*Reference*	0.204	0.543	0.087

Note: Chi-square was used. All comparisons were performed using no calcification as control.

All SNPs were in Hardy-Weinberg Equilibrium. Table 4 presents the allelic distribution according to the groups. The allele C of the *rs10177996* in *WNT10A* was associated with molar calcifications (p = 0.042) and with upper first molar calcification (p = 0.035), in which patients carrying the C allele had a lower risk to present dental pulp calcification. No other alleles were associated with molar calcifications.

**Table 4:** Allelic distribution o of *WNT10A* and *WNT6* genes polymorphisms.

SNP	Phenotype	MA* frequency (%)	p-value	OR (95% CI)
rs7349332 (Minor allele T)	Control (no calcification)	13.77	*Reference*	*Reference*
	Molar (upper + lower) calcification	11.54	0.607	0.82 (0.38–1.77)
	Upper molar calcification	10.87	0.479	0.75 (0.34–1.67)
	Lower molar calcification	12.00	0.847	0.91 (0.35–2.36)
rs3806557 (Minor allele A)	Control (no calcification)	24.24	*Reference*	*Reference*
	Molar (upper + lower) calcification	21.43	0.206	0.67 (0.36–1.24)
	Upper molar calcification	22.09	0.340	0.74 (0.39–1.38)
	Lower molar calcification	18.75	0.161	0.57 (0.26–1.26)
rs10177996 (Minor allele C)	Control (no calcification)	37.50	*Reference*	*Reference*
	Molar (upper + lower) calcification	24.45	**0.042**	0.60 (0.33–0.98)
	Upper molar calcification	24.49	**0.035**	0.55 (0.31–0.96)
	Lower molar calcification	26.92	0.640	0.85 (0.44–1.64)
rs6754599 (Minor allele A)	Control (no calcification)	18.18	*Reference*	*Reference*
	Molar (upper + lower) calcification	11.76	0.177	0.60 (0.28–1.27)
	Upper molar calcification	12.22	0.289	0.66 (0.31–1.42)
	Lower molar calcification	8.33	0.129	0.44 (0.15–1.30)

*MA means Minor Allele.

**Bold** indicates a statistically significant difference (p < 0.05).

Table 5 shows the genotype distribution among groups. The *rs10177996* in *WNT10A* showed a statistically significant p-value for molar calcification (p = 0.027) and upper molar calcification (p = 0.040) in the recessive model. Also, the *rs3806557* showed a significant p-value in the recessive model in molar calcification (p = 0.046). No others genotype showed significant results.

**Table 5:** Genotype distribution of WNT10A and WNT6 genes polymorphisms.

SNP	Phenotype	Genotype distribution (%)			p-value		
					*additive*	*dominant*	*recessive*
rs7349332		TT	CT	CC			
	Control (no calcification)	0 (0.00)	19 (25.00)	50 (65.79)	*Reference*		
	Molar (upper + lower) calcification	0 (0.00)	12 (21.43)	40 (71.43)	0.578*	0.578	#
	Upper molar calcification	0 (0.00)	10 (20.00)	36 (72.00)	0.483*	0.483	#
	Lower molar calcification	0 (0.00)	6 (22.22)	19 (70.37)	0.731*	0.731	#
rs3806557		AA	AG	GG			
	Control (no calcification)	8 (10.53)	22 (28.95)	36 (47.37)	*Reference*		
	Molar (upper + lower) calcification	1 (1.79)	19 (33.93)	29 (51.79)	0.136	0.620	**0.046**
	Upper molar calcification	1 (2.00)	17 (34.00)	25 (50.00)	0.186	0.712	0.070
	Lower molar calcification	0 (0.00)	9 (33.33)	15 (55.56)	0.202	0.500	0.074
rs10177996		CC	CT	TT			
	Control (no calcification)	9 (11.84)	36 (47.37)	27 (35.53)	*Reference*		
	Molar (upper + lower) calcification	1 (1.79)	26 (46.43)	28 (50.00)	0.053	0.131	**0.027**
	Upper molar calcification	1 (2.00)	22 (44.00)	26 (52.00)	0.060	0.090	**0.040**
	Lower molar calcification	1 (3.70)	12 (44.44)	13 (48.15)	0.334	0.266	0.212
rs6754599		GG	GC	CC			
	Control (no calcification)	3 (3.95)	18 (23.68)	45 (59.21)	*Reference*		
	Molar (upper + lower) calcification	0 (0.00)	12 (21.43)	39 (69.24)	0.253	0.323	0.123
	Upper molar calcification	0 (0.00)	11 (22.00)	34 (68.00)	0.312	0.400	0.147
	Lower molar calcification	0 (0.00)	4 (14.81)	20 (74.07)	0.292	0.156	0.288

* Null values were excluded from the analysis.

# Recessive p-values were not applicable by the null recessive frequencies.

**Bold** indicates a statistically significant difference (p < 0.05).

Haplotypes of the four SNPs were analyzed and are fully presented in S1 Table. Table 6 presents the haplotype frequency comparisons between the SNPs with significant statistically results.

**Table 6:** Haplotypes that were statistically associated for the studied SNPs from genes WNT10A and WNT6.

Haplotype		Frequency (%)		p-value
		Control	Case	
Molar (upper + lower) calcification				
rs6754599 | rs7349332 | rs3806557 | rs10177996	CCGT	0.50	0.66	**0.015**
rs6754599 | rs7349332 | rs380655	CCG	0.67	0.79	**0.045**
rs6754599 | rs7349332 | rs10177996	CCT	0.58	0.71	**0.030**
rs6754599 | rs10177996 | rs3806557	CTG	0.48	0.65	**<0.001**
rs6754599 | rs10177996	CT	0.57	0.70	**0.037**
Upper molar calcification				
rs6754599 | rs7349332 | rs3806557 | rs10177996	CCGT	0.51	0.67	**0.014**
rs6754599 | rs7349332 | rs10177996	CCT	0.58	0.73	**0.016**
rs6754599 | rs10177996 | rs3806557	CTG	0.49	0.66	**0.008**
rs6754599 | rs10177996	CT	0.57	0.72	**0.023**

Note: no statistically significant p-value was identified for lower molar calcification.

**Bold** indicates a statistically significant difference (p < 0.05).

## Discussion

The results showed associations between dental pulp calcification and the studied SNPs (in genotype, allelic and haplotype analyzes), confirming the central hypothesis of the study. Two main types of dental pulp calcification identified in clinical practice by radiographs are the pulp space narrowing and the pulp stone [5]. They appear to be different in terms of morphology and clinical implications [9]. The key differences between them are the clinical appearance, in which the pulp stone is presented as well-defined radiopaque masses and the pulp space narrowing as a diffuse calcification along the dentin walls, with or without projections [5]. The distribution of this calcifications varies widely, depending specially on the population and the method of analysis [9], but occurs mostly in molars [31]. It appears that both, pulp space narrowing and pulp stone, are physiological manifestation and may increase in volume and number due to an associated pathology [9].

SNPs are the most common type of genetic variation in humans. It is a type of genetic variant that involves a variation of a single base pair in the genome [32] and is associated with a variety of conditions, including endodontic [33–35] and orthodontic phenotypes [21, 22, 24, 27, 33]. It has been stated that these variants could determinate differences in individual predisposition, and even particularly different phenotypes on a similar population [36]. SNP’s near, or in, the WNT pathways genes have been previously reported to be related to critical regulation in bone homeostasis, bone mineral density, bone-related diseases [37] and dental-related phenotypes [21, 22, 27]. The results of this study show that SNPs in the WNT pathway are potentially related to the dentin-pulp complex mineral metabolism.

The canonical pathway has been related to dentin accumulation, in mice overexpressing β-catenin [3]. However, the findings indicated cells secrete dentin matrix in a rapid process, forming an atubular osteodentin structure-like. This remarks the WNT/ β-catenin involvement in dentin formation. The WNT10A is suggested to be involved in the dentin repair process, as it is especially expressed in the early stages of dentin formation [38]. The SNP rs10177996 in *WNT10A* seems to be related to overexpression of dentin mineral content, when we interpreted the results. Similar finds were described within *WNT7b* overexpressed [39], which caused a higher ratio of Ca/P in dentin, smaller tubule diameter, and fewer tubules as well. The opposite way was also stated, pointing that mutations in β-catenin, essential to the WNT canonical pathway, leads to defective odontoblast differentiation, thin dentinal walls, and disturbs dentinogenesis and cementogenesis [40].

The non-canonical WNT pathway regulates different physiological events, acting independently of β-catenin, in contrast to the canonical pathway. When WNT binds to its receptor, calcium is released intracellularly. This WNT/calcium pathway is associated with developmental processes [13]. In our study, we selected SNPs in the candidate genes *WNT10A* and *WNT6*. *WNT6*, one of the proteins in the non-canonical pathway, showed statistically significant results of the SNP rs6754599 only in the haplotype analysis. This indicates that this SNP could be a biomarker for dental pulp calcifications, however, more studies are necessary to confirm our results. Future studies should consider identifying the role of this gene and others related to the non-canonical WNT pathway. SNPs in *WNT10A* were associated with pulp calcification in the allele, genotype, and haplotype analysis, suggesting that SNPs in this gene are a candidate for dental pulp calcification and should be further explored by independent research groups. This is a promising field of investigation considering the intimate relation between developmental processes and mineral content regulation.

The dentin-pulp complex response to trauma must be considered, as the WNT pathway is also related to pulp innervation, enzymatic activity, and dental pulp inflammation that could lead to dentin repair and dentin bridges formation [3, 20]. The orthodontic movement is a stress factor to the pulp, which causes dental pulp inflammation in many degrees [8]. This injury could lead mesenchymal stem cells contained in the dental pulp to activate a differentiation to odontoblast-like cells and secreting dentin matrix. WNT signaling plays a significant role in tertiary dentin formation, as reported previously in pulp injury models [41]. This implies the involvement of the WNT canonical pathway in reparative dentin formation from odontoblast-like cell differentiation, in agreement with other studies [3].

Nevertheless, the role of WNT signaling in dentin deposition rate [3] is clinically relevant. Dental pulp injury could occur in different situations, iatrogenic or therapeutic. Orthodontic movement is one of the most performed dentistry-related procedures [8], and the pulp injury that it causes is collateral damage not fully controlled by the clinician.

Although the orthodontic treatment characteristics such as age of the patients, duration of the treatment and type of skeletal malocclusion were not associated with dental pulp calcification in our study, there are other orthodontic factors that could influence the phenotype. Orthodontic force is well known involved in dental pulp calcification [8], however, this is a difficult parameter to measure and to include in the study design. It is also possible that differences in the fixed orthodontic treatment may be involved in the risk of dental pulp calcification. These are limitations of our study once these factors were not evaluated.

Other limitations of our study should be highlighted here. This study used panoramic radiographs to identify pulp calcification. The literature indicates that the radiographic exams are the most used for the assessment of this condition, but it also points to some disadvantages, like limited resolution, impossibility to identify calcifications lesser than 200°m and structure overlap [42]. Previous studies used Cone-beam computerized tomography (CBCT) and Microcomputed tomography (MicroCT) as tools to assess pulp calcifications, once these exams are more precise than radiographs [31, 42]. However, CBCT and MicroCT were not used in our study once the panoramic radiographs were taken as follow-up images for the orthodontic treatment and, therefore, additional image exams were not performed for this study in order to protect the patients from additional exposure. Due to the limitation of the 2D exams, we decided to group pulp stones and narrowing of the radicular pulp space as a single phenotype. Although the absence of stratification of the pulp calcification phenotypes could be also a limitation, our hypothesis is that WNT10A and WNT6 act in both phenotypes in a similar fashion. WNT10A and WNT6 have an important role in dentin secretion and dentin bridge formation [20].

This study is limited to the SNPs analyzed. However, it highlights the potential of other WNT pathway as biomarkers for dental pulp response to injury, dentin formation, and dental pulp calcification that could allow the identification of patients with a higher risk to develop dental pulp calcification as an adverse sequela of orthodontic treatment.

Strong evidences suggest the role of WNT pathway as the foundation for regenerative dentistry in the future [12], and it must be further investigated also as biomarker for dental traits. Future studies should investigate the main role of the WNT canonical and non-canonical pathways in dentinogenesis, from tooth formation to tertiary or reparative dentin secretion. The comprehension of predisposing factors, such as the SNPs, could lead to a better understanding of the aspects involved in dental pulp calcification after orthodontic treatment. The knowledge of the clinical parameters and molecular factors involved in dental pulp calcification after orthodontic treatment will allow the prediction of high-risk patients. Allowing a personalized treatment plan and reducing the collateral damage to the pulp complex. Other SNPs in the WNT pathway as well, mainly those aroused in the pulp-dentin complex mineral metabolism and mesenchymal stem cell signaling and differentiation should be further studied to identify a panel of possible biomarkers for dental pulp calcification. It is possible that a combination of many biomarkers, as is observed in other oral conditions [43], will aid the identification of patients with higher risk for pulp calcification after orthodontic treatment.

## Conclusions

The findings of this study indicates that SNPs in *WNT10A* and *WNT6* are associated with dental pulp calcification of molars after orthodontic dental treatment and may be considered as biomarkers for dental pulp calcification.

## Supporting information

S1 File (DOCX)

S1 Table Full haplotype analysis of the study’s genetic polymorphisms from genes WNT10A and WNT6. (DOCX)

S1 Checklist STrengthening the REporting of Genetic Association studies (STREGA) reporting recommendations, extended from STROBE statement. (DOCX)
