# Correction: The investigation of *WNT6* and *WNT10A* single nucleotide polymorphisms as potential biomarkers for dental pulp calcification in orthodontic patients

**DOI:** 10.1371/journal.pone.0305377

**Published:** 2024-06-06

**Authors:** Iago Ramirez, Christian Kirschneck, Alice C Silva-Sousa, Peter Proff, Leonardo S. Antunes, Marilisa CL Gabardo, Daniela Silva Barroso de Oliveira, Manoel D. Sousa-Neto, Flares Baratto-Filho, Erika C. Küchler

The third, sixth and seventh authors name are incorrectly written. The correct names are: Alice C Silva-Sousa, Marilisa CL Gabardo and Daniela SB de Oliveira respectively. The correct citation is: Ramirez I, Kirschneck C, Silva-Sousa AC, Proff P, S. Antunes L, Gabardo MCL, et al. (2023) The investigation of WNT6 and WNT10A single nucleotide polymorphisms as potential biomarkers for dental pulp calcification in orthodontic patients. PLOS ONE 18(8): e0288782. https://doi.org/10.1371/journal.pone.0288782.
