# Supplementary material for: The investigation of WNT6 and WNT10A single nucleotide polymorphisms as potential biomarkers for dental pulp calcification in orthodontic patients
Source: PLoS One. 2023 Aug 11;18(8):e0288782. doi: 10.1371/journal.pone.0288782 (PMC10420345; doi:10.1371/journal.pone.0288782)
Supplement: S1 File — (DOCX) [file pone.0288782.s001.docx]

The whole data is available at: Link: <https://osf.io/8np9t/>
