# Supplementary material for: The investigation of WNT6 and WNT10A single nucleotide polymorphisms as potential biomarkers for dental pulp calcification in orthodontic patients
Source: PLoS One. 2023 Aug 11;18(8):e0288782. doi: 10.1371/journal.pone.0288782 (PMC10420345; doi:10.1371/journal.pone.0288782)
Supplement: S1 Table — (DOCX) [file pone.0288782.s002.docx]

**Supplementary material**

| **Supplementary Table 1.** Full haplotype analysis of the study’s genetic polymorphisms from genes WNT10a and WNT6. | | | | |
| --- | --- | --- | --- | --- |
| **Haplotype** | | **Frequency (%)** | | **p-value** |
|  |  | **Control** | **Case** |  |
| Molar (upper + lower) calcification | | | | |
| rs6754599 \| rs7349332 \| rs3806557 \| rs10177996 | GTAC | 0.07 | 0.05 | 0.360 |
|  | CTAC | 0.02 | 0.03 | 0.898 |
|  | GCAC | 0.04 | 0.03 | 0.601 |
|  | CCAC | 0.04 | 0.01 | 0.122 |
|  | CCGC | 0.18 | 0.13 | 0.362 |
|  | GTAT | 0.03 | 0.04 | 0.816 |
|  | CCAT | 0.06 | 0.05 | 0.784 |
|  | GCGT | 0.04 | <0.01 | 0.060 |
|  | CCGT | 0.50 | 0.66 | **0.015** |
| rs6754599 \| rs7349332 \| rs3806557 | GTA | 0.11 | 0.08 | 0.538 |
|  | CTA | 0.03 | 0.03 | 0.830 |
|  | GCA | 0.04 | 0.03 | 0.707 |
|  | CCA | 0.11 | 0.06 | 0.240 |
|  | GCG | 0.04 | <0.01 | 0.070 |
|  | CCG | 0.67 | 0.79 | **0.045** |
| rs6754599 \| rs7349332 \| rs10177996 | GTC | 0.08 | 0.05 | 0.383 |
|  | CTC | 0.02 | 0.03 | 0.886 |
|  | GCC | 0.04 | 0.02 | 0.440 |
|  | CCC | 0.22 | 0.14 | 0.153 |
|  | GTT | 0.03 | 0.03 | 0.756 |
|  | GCT | 0.03 | <0.01 | 0.143 |
|  | CCT | 0.58 | 0.71 | **0.030** |
| rs6754599 \| rs10177996 \| rs3806557 | GCA | 0.11 | 0.08 | 0.315 |
|  | CCA | 0.07 | 0.03 | 0.198 |
|  | GTA | 0.02 | 0.04 | 0.547 |
|  | CTA | 0.08 | 0.06 | 0.693 |
|  | CCG | 0.19 | 0.13 | 0.250 |
|  | GTG | 0.04 | <0.01 | 0.091 |
|  | CTG | 0.48 | 0.65 | **<0.001** |
| rs7349332 \| rs3806557 \| rs10177996 | TAC | 0.09 | 0.07 | 0.572 |
|  | CAC | 0.08 | 0.04 | 0.125 |
|  | CGC | 0.18 | 0.14 | 0.360 |
|  | TAT | 0.04 | 0.04 | 0.929 |
|  | CAT | 0.06 | 0.06 | 0.946 |
|  | CGT | 0.54 | 0.66 | 0.066 |
| rs6754599 \| rs7349332 | GT | 0.10 | 0.09 | 0.656 |
|  | CT | 0.03 | 0.03 | 0.966 |
|  | GC | 0.08 | 0.03 | 0.132 |
|  | CC | 0.78 | 0.85 | 0.208 |
| rs3806557 \| rs10177996 | AC | 0.18 | 0.11 | 0.148 |
|  | GC | 0.18 | 0.14 | 0.429 |
|  | AT | 0.10 | 0.10 | 0.919 |
|  | GT | 0.53 | 0.64 | 0.087 |
| rs7349332 \| rs3806557 | TA | 0.15 | 0.12 | 0.648 |
|  | CA | 0.15 | 0.10 | 0.237 |
|  | CG | 0.70 | 0.78 | 0.213 |
| rs6754599 \| rs10177996 | GC | 0.12 | 0.07 | 0.231 |
|  | CC | 0.25 | 0.18 | 0.208 |
|  | GT | 0.06 | 0.04 | 0.574 |
|  | CT | 0.57 | 0.70 | **0.037** |
| rs7349332 \| rs10177996 | TC | 0.10 | 0.07 | 0.532 |
|  | CC | 0.24 | 0.17 | 0.163 |
|  | TT | 0.04 | 0.04 | 0.977 |
|  | CT | 0.61 | 0.71 | 0.116 |
| Lower molar calcification | | | | |
| rs6754599 \| rs7349332 \| rs3806557 \| rs10177996 | GTAC | 0.07 | 0.03 | 0.340 |
|  | CTAC | 0.02 | 0.06 | 0.120 |
|  | GCAC | 0.04 | 0.02 | 0.503 |
|  | CCAC | 0.04 | <0.01 | 0.180 |
|  | CCGC | 0.16 | 0.13 | 0.560 |
|  | GTAT | 0.03 | 0.02 | 0.724 |
|  | CCAT | 0.06 | 0.04 | 0.609 |
|  | GCGT | 0.03 | <0.01 | 0.265 |
|  | CCGT | 0.54 | 0.69 | 0.070 |
| rs6754599 \| rs7349332 \| rs3806557 | GTA | 0.11 | 0.06 | 0.321 |
|  | CTA | 0.02 | 0.06 | 0.220 |
|  | GCA | 0.04 | 0.02 | 0.638 |
|  | CCA | 0.10 | 0.04 | 0.186 |
|  | GCG | 0.03 | <0.01 | 0.268 |
|  | CCG | 0.70 | 0.81 | 0.116 |
| rs6754599 \| rs7349332 \| rs10177996 | GTC | 0.07 | 0.03 | 0.334 |
|  | CTC | 0.02 | 0.06 | 0.119 |
|  | GCC | 0.04 | 0.02 | 0.454 |
|  | CCC | 0.20 | 0.13 | 0.277 |
|  | GTT | 0.03 | 0.02 | 0.774 |
|  | GCT | 0.03 | <0.01 | 0.355 |
|  | CCT | 0.61 | 0.73 | 0.124 |
| rs6754599 \| rs10177996 \| rs3806557 | GCA | 0.11 | 0.05 | 0.213 |
|  | CCA | 0.06 | 0.04 | 0.682 |
|  | GTA | 0.03 | 0.03 | 0.954 |
|  | CTA | 0.08 | 0.06 | 0.624 |
|  | CCG | 0.17 | 0.17 | 0.890 |
|  | GTG | 0.03 | <0.01 | 0.276 |
|  | CTG | 0.53 | 0.64 | 0.147 |
| rs7349332 \| rs3806557 \| rs10177996 | TAC | 0.08 | 0.08 | 0.986 |
|  | CAC | 0.08 | 0.01 | 0.108 |
|  | CGC | 0.16 | 0.17 | 0.860 |
|  | TAT | 0.04 | 0.03 | 0.650 |
|  | CAT | 0.06 | 0.04 | 0.662 |
|  | CGT | 0.58 | 0.65 | 0.294 |
| rs6754599 \| rs7349332 | GT | 0.10 | 0.06 | 0.336 |
|  | CT | 0.02 | 0.06 | 0.170 |
|  | GC | 0.07 | 0.02 | 0.227 |
|  | CC | 0.80 | 0.85 | 0.405 |
| rs3806557 \| rs10177996 | AC | 0.17 | 0.10 | 0.247 |
|  | GC | 0.16 | 0.18 | 0.751 |
|  | AT | 0.11 | 0.08 | 0.522 |
|  | GT | 0.56 | 0.64 | 0.317 |
| rs7349332 \| rs3806557 | TA | 0.14 | 0.12 | 0.782 |
|  | CA | 0.14 | 0.06 | 0.146 |
|  | CG | 0.72 | 0.81 | 0.191 |
| rs6754599 \| rs10177996 | GC | 0.11 | 0.05 | 0.210 |
|  | CC | 0.22 | 0.20 | 0.683 |
|  | GT | 0.06 | 0.03 | 0.447 |
|  | CT | 0.60 | 0.72 | 0.138 |
| rs7349332 \| rs10177996 | TC | 0.09 | 0.09 | 0.968 |
|  | CC | 0.23 | 0.15 | 0.215 |
|  | TT | 0.04 | 0.03 | 0.786 |
|  | CT | 0.63 | 0.72 | 0.226 |
| Upper molar calcification | | | | |
| rs6754599 \| rs7349332 \| rs3806557 \| rs10177996 | GTAC | 0.07 | 0.05 | 0.606 |
|  | CTAC | 0.03 | 0.02 | 0.694 |
|  | GCAC | 0.04 | 0.03 | 0.817 |
|  | CCAC | 0.04 | 0.01 | 0.195 |
|  | CCGC | 0.18 | 0.12 | 0.171 |
|  | GTAT | 0.03 | 0.03 | 0.829 |
|  | CCAT | 0.06 | 0.06 | 0.908 |
|  | GCGT | 0.03 | <0.01 | 0.096 |
|  | CCGT | 0.51 | 0.67 | **0.014** |
| rs6754599 \| rs7349332 \| rs3806557 | GTA | 0.10 | 0.08 | 0.580 |
|  | CTA | 0.04 | 0.02 | 0.501 |
|  | GCA | 0.03 | 0.03 | 0.936 |
|  | CCA | 0.10 | 0.07 | 0.481 |
|  | GCG | 0.03 | <0.01 | 0.108 |
|  | CCG | 0.68 | 0.78 | 0.096 |
| rs6754599 \| rs7349332 \| rs10177996 | GTC | 0.07 | 0.05 | 0.634 |
|  | CTC | 0.03 | 0.02 | 0.706 |
|  | GCC | 0.04 | 0.03 | 0.623 |
|  | CCC | 0.22 | 0.13 | 0.081 |
|  | GTT | 0.03 | 0.03 | 0.877 |
|  | GCT | 0.03 | <0.01 | 0.210 |
|  | CCT | 0.58 | 0.73 | **0.016** |
| rs6754599 \| rs10177996 \| rs3806557 | GCA | 0.10 | 0.08 | 0.614 |
|  | CCA | 0.07 | 0.03 | 0.177 |
|  | GTA | 0.02 | 0.03 | 0.862 |
|  | CTA | 0.07 | 0.07 | 0.870 |
|  | CCG | 0.20 | 0.12 | 0.106 |
|  | GTG | 0.04 | <0.01 | 0.120 |
|  | CTG | 0.49 | 0.66 | **0.008** |
| rs7349332 \| rs3806557 \| rs10177996 | TAC | 0.09 | 0.07 | 0.606 |
|  | CAC | 0.08 | 0.04 | 0.248 |
|  | CGC | 0.19 | 0.12 | 0.175 |
|  | TAT | 0.05 | 0.03 | 0.621 |
|  | CAT | 0.05 | 0.06 | 0.747 |
|  | CGT | 0.54 | 0.67 | 0.054 |
| rs6754599 \| rs7349332 | GT | 0.10 | 0.09 | 0.718 |
|  | CT | 0.04 | 0.02 | 0.560 |
|  | GC | 0.07 | 0.03 | 0.253 |
|  | CC | 0.79 | 0.85 | 0.230 |
| rs3806557 \| rs10177996 | AC | 0.17 | 0.12 | 0.270 |
|  | GC | 0.19 | 0.12 | 0.214 |
|  | AT | 0.10 | 0.10 | 0.947 |
|  | GT | 0.53 | 0.65 | 0.075 |
| rs7349332 \| rs3806557 | TA | 0.15 | 0.12 | 0.542 |
|  | CA | 0.13 | 0.10 | 0.557 |
|  | CG | 0.72 | 0.77 | 0.359 |
| rs6754599 \| rs10177996 | GC | 0.11 | 0.08 | 0.498 |
|  | CC | 0.26 | 0.16 | 0.084 |
|  | GT | 0.06 | 0.03 | 0.424 |
|  | CT | 0.57 | 0.72 | **0.023** |
| rs7349332 \| rs10177996 | TC | 0.10 | 0.07 | 0.584 |
|  | CC | 0.25 | 0.16 | 0.112 |
|  | TT | 0.04 | 0.03 | 0.679 |
|  | CT | 0.61 | 0.72 | 0.060 |
| **Bold** indicates a statistically significant difference (p < 0.05). | | | | |
|  | | | | |
